# The Distribution Frequency of Interferon-Gamma Receptor 1 Gene Polymorphisms in Interferon-*γ* Release Assay-Positive Patients

**DOI:** 10.1155/2017/4031671

**Published:** 2017-10-25

**Authors:** Changguo Chen, Lei Chen, Changwei Chen, Qiuyuan Chen, Qiangyuan Zhao, Youyou Dong

**Affiliations:** ^1^Department of Clinical Laboratory, The Navy General Hospital, No. 6 Fucheng Road, Beijing 100037, China; ^2^Department of Pathology, Donghua Hospital Affiliated to Zhongshan University, No. 1 Dongcheng Road, Dongguan, Guangdong 523110, China

## Abstract

Tuberculosis is caused by mycobacterium, a potentially fatal infectious bacterium. In recent years, TB cases increased in the whole world. WHO statistics data shows that the world's annual tuberculosis incidence was 8~10 million with about 3 million deaths. Several studies have shown that susceptibility to tuberculosis may be associated with IFNGR1 gene polymorphisms. Here, we report the distribution frequency of IFNGR1 gene polymorphisms in 103 cases of IGA-negative patients and 100 cases of IGA-positive patients from China by sequencing the IFNGR1 proximal ~750 bp promoter region. We found a total of 5 types of site mutations: -611 (G/A), -56 (T/C), -255 (C/T), -359 (T/C), and -72 (C/T). The two main types of gene polymorphisms among the IGA-negative and IGA-positive groups were -611 (G/A), with mutation rates of 88.3% and 78.4%, respectively, and -56 (T/C), with mutation rates of 84.5% and 83.8%, respectively, which had no statistical significance, and there was no correlation with the incidence of tuberculosis.

## 1. Background

As a common infectious disease, the prevalence of tuberculosis (TB) has been increasing around the globe, especially in Asia and Africa, and is influenced by multiple factors [1–3]. WHO statistics data demonstrate that the world's annual tuberculosis incidence was 8~10 million and approximately 3 million people died of tuberculosis, which is the largest number of deaths caused by a single infectious disease [4–6]. In the past 10 years, the incidence of bacteriologically positive pulmonary tuberculosis patients in our country was less than 30%, as evidenced from the results of the digital epidemiological survey conducted every year [4, 5]. China is one of the 22 countries with the highest burden of TB in the world, ranking second in the number of TB patients, after India [7–10]. In addition to environmental factors, an increasing number of studies and reviews indicate that host genetic factors play an important role in susceptibility to tuberculosis [6, 11–13].

The IFN-*γ* release assay (IGRA) is an in vitro release enzyme-linked immunosorbent assay used to measure specific antigen-mediated cellular immune responses. A *Mycobacterium tuberculosis*-specific recombinant antigen was used to stimulate specific T lymphocytes and make them proliferate. The plasma levels of IFN-*γ* released by the sensitized T cells in the whole blood, after being stimulated by the MTB-specific antigen, were detected to determine *Mycobacterium tuberculosis* infection. IGRAs can offset the drawbacks of tuberculin skin testing [14–17]. At present, several countries routinely diagnose MTB latent infections (latent tuberculosis infection (LTBI)), which has a positive significant effect on tuberculosis control.

IFN-*γ* plays an important role in innate and adaptive immunity and was first discovered in 1965; in addition, it belongs to the type II interferon. IFNGR1 is the IFN-*γ* receptor *α* chain; it is expressed on the surface of all cells except red blood cells and is necessary for the binding of IFN-*γ* and its signal transduction [18, 19]. The SNP of the IFNGR1 promoter has been associated with various diseases. Several studies have demonstrated that susceptibility to tuberculosis may be associated with IFNGR1 gene polymorphisms [20–22]. Thus, the present study aimed to examine the distribution frequency of interferon-gamma (IFN-*γ*) receptor 1 (IFNGR1) gene polymorphisms in patients with positive interferon-*γ* release assays.

## 2. Methods

### 2.1. General Data

A total of 203 hospitalized patients who were suspected of tuberculosis infection by clinicians at the Navy General Hospital from March 2015 to April 2016 were enrolled in the research group, including 103 cases of IFN-*γ* release assay result-negative patients and 100 cases of IFN-*γ* release assay result-positive patients. The patients' basic information, results of tuberculin skin testing and results of the Zeihl-Neelsen acid-fast stain test, were collected from the hospital's digital medical record system. Overall, there were 136 males within the age range of 19 to 93 years and 67 females within the age range of 23 to 87 years.

### 2.2. Detecting Plasma Levels of IFN-*γ*

Venous puncture was used to collect the whole blood samples, no less than 4 mL. The blood samples were gently mixed evenly 3~5 times and then divided into three groups of culture tubes: N (control), T (test), and P (positive control); 1 mL/tube. The cultivation pipes were gently mixed upside down 5 times and incubated for 22 ± 2 hours at 37°C. Following incubation, the culture tubes were centrifuged for 10 minutes at 3000~5000 rpm/min. Fifty microliters of supernatant was used to detect IFN-*γ* levels by ELISA, and the cutoff value was >14 pg/mL, and interpretation of the results is presented in Table 1.

### 2.3. Extraction of Genomic DNA

Total genomic DNA of leukocytes was extracted from 0.2 mL of peripheral blood using the Whole Blood DNA Extraction Kit (Tiangen Biotech Co. Ltd., Beijing, China), according to the manufacturer's instructions. The genomic DNA extracted was dissolved in 0.1x TE buffer (10 mM Tris −1 mM EDTA, pH 8.0) and stored at −20°C.

### 2.4. Amplification and Sequencing of IFNGR1 Proximal Promoter PCR

Primer premier 5.0 software was used to design primers for amplification of 750 bp proximal fragments of IFNGR1; forward 5′- CAGGTGAGATCATTAGACATTCGC-3′ and reverse 5′- GCTGCTACCGACGGTCGCTG -3′. In each 0.2 mL PCR reaction tube, 1 *μ*L of genomic DNA (100 ng/mL), 1 *μ*L of each primer, 10 *μ*L of 2 X PrimeTaq Premix (Tiangen Biotech, Co., Ltd, Beijing, China), and the appropriate amount of ddH2O were added. The PCR cycling conditions were as follows: initial denaturation at 95°C for 5 min followed by 30 cycles for 30 s at 94°C, annealing at 58°C for 30 s, and extension at 72°C for 45 s, with a final extension at 72°C for 7 min. The PCR products were run on 1.0% agarose gels containing 0.5% GoldView™ and observed under UV light. The genotyping of the IFNGR1 sequence was done by Sanger sequencing (Aoke DINGSHENG Biotechnology Co. Ltd., Beijing, China).

### 2.5. Statistical Analysis

Analysis of data was done using the SPSS 19.0 software. Differences between variables were evaluated by Fisher's exact test according to the data. The associations between genotypes and IFN-*γ* levels were calculated using the Cochran-Armitage test for trend. *P* values less than 0.05 were considered statistically significant.

## 3. Results

### 3.1. Characteristics of Patients

The study consisted of 103 IGA-negative patients (70 males aged 23 to 88 years and 33 females aged 23 to 90 years), and pulmonary disease was detected in 76 cases of 100 IGA-positive patients (66 males aged 19 to 93 years and 34 females aged 24 to 87 years). Pulmonary disease was detected in 55 cases, of which 2 cases were diagnosed as tuberculosis. There were 82 cases that were purely IGA-positive and 18 cases of positive evidence of other TB-related indicators; 15 cases were positive for IGA and tuberculin skin testing; 1 case was positive for IGA and the Zeihl-Neelsen acid-fast stain test; 2 cases were positive for IGA, Tuberculin skin testing and the Zeihl-Neelsen acid-fast stain test (Table 2). There was no significant difference between the groups with respect to sex and age (*P* > 0.05).

### 3.2. Mutations Obtained by BioEdit Sequence Alignment

**Figure 1:**
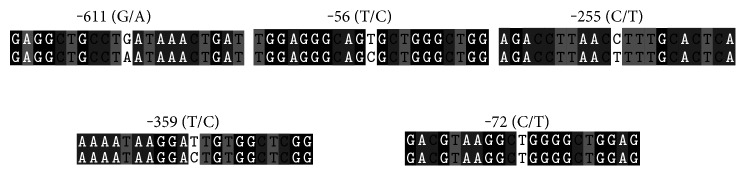


Sequence information was obtained by first generation sequencing technology. In the IGA-negative group, the -611 (G/A) mutation rate was 88.3%, and the -56 (T/C) mutation rate was 84.5% (-611 (G/A), -56 (T/C): 79 cases; -611 (G/A), -56 (T/T): 14 cases; -611 (G/G), -56 (T/C): 9 cases; and -611 (G/G), -56 (T/T): 3 cases). In the IGA-positive group, the -611 (G/A) mutation rate was 78% and the -56 (T/C) mutation rate was 83% (-611 (G/A), -56 (T/C): 70 cases; -611 (G/A), -56 (T/T): 8 cases; -611 (G/G), -56 (T/C): 13 cases; and -611 (G/G), -56 (T/T): 9 cases). Other mutations were also observed, such as -255 (C/T), -359 (T/C), and -72 (C/T) (Figure 1).

### 3.3. Distribution of the IFNGR1 Promoter Mutation in Different Plasma Levels of IFN-*γ*

According to the plasma levels of IFN-*γ*, the subjects were divided into four groups: IFN-*γ* > 400 pg/mL, 200 pg/mL < IFN-*γ* < 400 pg/mL, IFN-*γ* < 200 pg/mL, and IFN-*γ* < 14 pg/mL. The mutations in the IFNGR1 gene were observed at different loci corresponding to different IFN-*γ* levels, and the mutations -611 (G/A) and -56 (T/C) were the highest among the gene polymorphisms of the IFNGR1 promoter but were uncorrelated with the plasma levels of IFN-*γ*, *P* > 0.005 (Table 3).

## 4. Discussion

Tuberculosis, which is an infectious disease and remains a major public health problem as well as a leading cause of morbidity, has plagued human beings for thousands of years [1, 6, 23]. In recent years, the incidences and mortality of tuberculosis have been rising. Infection with *Mycobacterium tuberculosis* is related not only to the external environment but also to the impact of genetic factors on the phenotypic variation and immune responses in the population infected with TB [24–27]. Although the evidence for a human genetic component in susceptibility to TB is incontrovertible, some genetic variation in cytokine-associated genes, including IFNGR1 and IFNGR2, has previously been found to be important in other viral/host-mediated immune responses in TB [28, 29]. IFNGR1, as a key molecule of the IFN-*γ* signalling pathway, was believed to play a key role in the pathogenesis of TB. People with hereditary IFNGR1 disorder are more susceptible to TB [30–32]. Shin et al. suggested that certain genetic variants in IFNGR genes may be associated with TB development [33]; Lü et al. demonstrated that rs2234711, rs1327475, and rs7749390 polymorphisms of the IFNGR1 gene were significantly associated with the altered risks of TB [21]. IFNGR1 proximal promoter gene polymorphisms also were believed that they were positively associated with an increased susceptibility to *Mycobacterium leprae* [34] and that IFNGR1-56 T/C polymorphism was a “biomarker” for identifying populations at higher risk of *Nontuberculous mycobacteria* infection [35]. However, Bulat-Kardum et al. noted that there was no significant correlation between the susceptibility of tuberculosis with the IFNGR1 proximal promoter -611G/A and -56 T/C gene polymorphisms [36]. Furthermore, Rosenzweig et al. suggested that the -611G/A and -56 T/C gene polymorphisms were not associated with increased mycobacterial susceptibility [37]. The meta-analysis of Wang et al. believed that IFNGR1 -56C/T is possibly associated with increased TB risk in Africans, but not in Asians or Caucasians [38].

In the present study, we screened 103 patients of Chinese Han nationality who were IGA negative and 100 patients who were IGA positive. By sequencing the IFNGR1 promoter, we found a total of 5 types of site mutations: -611 (G/A), -56 (T/C), -255 (C/T), -359 (T/C), and -72 (C/T). The two main types of gene polymorphisms among the IGA-negative and IGA-positive groups were -611 (G/A), with mutation rates of 88.3% and 78.4%, respectively, and -56 (T/C), with mutation rates of 84.5% and 83.8%, respectively, which had no statistical significance. There were 9 cases with -255 (C/T) mutation, 1 case with -359 (T/C) mutation, and 1 case with -72 (C/T) mutation. Although the mutation rates were low, their potential clinical value needs to be further verified. Because we are not a hospital specializing in treating tuberculosis, the number of confirmed cases was relatively small. In 4 cases, the patients were Zeihl-Neelsen acid-fast stain positive, 1 patient had the -611 (G/A) and -56 (T/C) mutations, and 3 patients had the -56 (T/C) mutation. Of the 55 patients with pulmonary disease, the -611 (G/A) mutation was observed in 52 patients and the -56 (T/C) mutation was observed in 48 patients.

There are some limitations to our study, we observed that the -611 (G/A) and -56 (T/C) mutations in IGA-positive patients have no difference compared with the IGA-negative patients. Therefore, we believe that there is not a certain correlation between the incidence of the -611 (G/A) and -56 (T/C) mutations and TB.

## Figures and Tables

**Table 1:** 

*n*	P–N	T–N	Results	Result interpretation
≤400	Any value	≥14 and ≥N/4	Positive	*Mycobacterium tuberculosis* infection (tuberculosis activity stage infection, latent infection, or positive infection)
	≥20	<14	Negative	Uninfected *Mycobacterium tuberculosis*
	≥20	≥14 但 < N/4	Negative	
	<20	<14	Not sure	It is not certain whether the *Mycobacterium tuberculosis* infection is infected
	<20	≥14 但 < N/4	Not sure	
>400	Any value	Any value	Not sure	

**Table 2:** Characteristics of 100 IGA-positive patients.

Characteristics	Number
Patients	100
Male/female	66/34
Age range	19–93/24–87
Pulmonary disease	55
Purely IGA positive	82
Positive evidence of other TB-related indicators	
IGA and tuberculin skin testing positive	15
IGA and the Zeihl-Neelsen acid-fast stain positive	1
IGA, tuberculin skin testing, and the Zeihl-Neelsen acid-fast stain positive	2

**Table 3:** Mutation of IFNGR1 promoter site in different IFN-*γ* l levels (*n* (%)).

IFN-*γ* level	*n*	-611 (G/A)	-56 (T/C)	-255 (C/T)	-359 (T/C)	-72 (C/T)
IFN-*γ* > 400 pg/mL	18	16 (89%)	16 (89%)	3 (11%)	1 (0.05%)	0 (0%)
200 pg/mL < IFN-*γ* < 400 pg/mL	28	21 (75%)	25 (89%)	0 (0%)	0 (0%)	0 (0%)
14 pg/mL < IFN-*γ* < 200 pg/mL	54	41 (76%)	42 (78%)	4 (0.07%)	0 (0%)	1 (0.02%)
IFN-*γ* < 14 pg/mL	103	91 (88.3%)	87 (84.5)	2 (1.9%)	0 (0%)	0 (0%)
